# Coronary atherosclerosis and periodontitis have similarities in their clinical presentation

**DOI:** 10.3389/froh.2023.1324528

**Published:** 2024-01-16

**Authors:** Marcelo Barbosa De Accioly Mattos, Camila Bernardo Peixoto, José Geraldo de Castro Amino, Leandro Cortes, Bernardo Tura, Martha Nunn, Marcia Giambiagi-deMarval, Carmelo Sansone

**Affiliations:** ^1^ School of Dentistry, Federal University of Rio de Janeiro, Rio de Janeiro, Brazil; ^2^ Division of Periodontics, University of Kentucky College of Dentistry, Lexington, KY, United States; ^3^ Department of Cardiology, Instituto Nacional de Cardiologia, Rio de Janeiro, Brazil; ^4^ Department of Biostatistic, Nunn Biostatistical Solutions, Omaha, NE, United States; ^5^ Instituto de Microbiologia Professor Paulo de Góes, Federal University of Rio de Janeiro, Rio de Janeiro, Brazil; ^6^ Departamento de Periodontia, Federal University of Rio de Janeiro, Rio de Janeiro, Brazil

**Keywords:** periodontitis, coronary heart disease, atherosclerosis, atheroma burden, inflammation

## Abstract

**Background:**

Periodontitis (PE) and coronary heart disease (CHD) possess multiple mechanisms for a putative association. This case-control study compared the periodontal status among CHD subjects to controls without CHD, while also investigating atheroma invasion by known periodontal pathogens.

**Methods:**

161 subjects participated in this study were divided into three CHD groups: No CHD, chronic CHD, acute CHD. Additional analysis involved grouping subjects according to number of atheromas: no atheroma, 1–4 atheromas, 5–18 atheromas. Data were collected from medical records, periodontal examinations, and questionnaires that included demographic, behavioral, and oral health variables. Angiographic catheterizations were analyzed according to the number of atheroma lesions, lesion size, lesion location, and atheroma lesion stability. Lipoprotein profile, inflammatory markers and cells were analyzed. The microbiological branch added 30 individuals who had their atheroma lesion and subgingival plaque analyzed using polymerase chain reaction probes against the 16 s region, red complex and *Aggregatibacter actinomycetemcomitans*' DNA.

**Results:**

Subjects with CHD had high levels of systemic inflammatory markers and low levels of high-density lipoproteins compared to subjects without CHD. Subjects without CHD and clear coronaries had a prevalence of mild CAL, while individuals with more atheroma lesions had advanced CAL and more active PE. Subjects with more advanced CAL were 4 times more likely to have CHD compared to subjects with less, which is comparable to smoking. Only 4 subjects had the screened pathogens detected in atheroma, although these subjects also have the screened pathogens in subgingival plaque. However, 80% of atheromas had bacteria.

**Conclusions:**

CHD and PE showed similarities in progression while active PE led to more atheroma lesions that also tended to be larger in size.

## 1 Introduction

For over a hundred years, oral diseases induced by bacteria—particularly periodontitis—have been potentially linked to fifty-seven diseases (1, 2). Due to the mortality rate of coronary heart disease (CHD) and the ease of diagnosing periodontitis (PE), the CHD-PE association became a critical area of scientific interest, resulting in numerous publications over the past three decades. Consensus was established between societies of periodontology and cardiology with top researchers from both fields investigating various putative pathways for a plausible relationship between these two diseases, following the establishment of strong epidemiological evidence of an association of these two disease entities (3, 4).

Briefly, advanced periodontitis generally leads to increased tooth loss, which, in turn, can result in dietary changes that relies on increased intake of lipids and carbohydrates, which is exactly the kind of diet frequently associated with CHD (5). Cytokines and bacteria from active periodontitis can reach the bloodstream with the potential to instigate or accelerate a variety of local and/or systemic pathological processes in the body, including the endothelial/sub-endothelial injury that results in the formation of atheroma lesions (5, 6).

Despite the cross-sectional nature of case-control studies, relevant characteristics of a population at a given time can still be ascertained through a carefully conducted study. The selection of an appropriate control group is also a challenging aspect of investigating the CHD-PE correlation, with publications usually relying on the clinical periodontal status and the cardiology diagnosis. In this manuscript, controls are composed of subjects without CHD, which was demonstrated not only by the clinical diagnosis, but also by the absence of atheroma lesions in the coronary arteries. Also, the correlation of the disease burden of both diseases, only briefly reported once (7), is one of the focuses of this manuscript.

This manuscript reports the similarities between the periodontal and coronary statuses. In this way, the relative clinical progression of periodontitis and the progression of coronary heart disease as exhibited by the number of coronary atheroma lesions can provide insight into the relative disease burden of PE and CHD. In addition, a short investigation into the presence of known periodontal pathogens inside atheroma lesions was performed.

## 2 Materials and methods

Participants were patients from the National Institute of Cardiology, Rio de Janeiro, Brazil. Data and sample collection were initiated upon approval from the hospital's committee for ethics in research (the Brazilian equivalent of the Institutional Review Board—IRB), under protocols 0018/26.11.2001 and 0025/23.12.2002, in accordance with the Helsinki declaration of 1975, as revised in 2013. Subjects agreed to participate after informed consents were obtained, which entailed providing all information about the procedures to be performed and any potential risks to the study subjects.

Participant selection—subjects with other inflammatory diseases, aside from coronary heart disease (CHD) and gingivitis/periodontitis (PE), were excluded. Subjects with dental implants, taking antibiotics, and unable to have an oral examination performed (e.g., patients using an endotracheal tube) were also excluded.

Subjects were classified into three groups based on the diagnosis for CHD: chronic coronary heart disease (CCHD), acute coronary heart disease (ACHD), and the non-coronary heart disease, or the control group (CG). 161 subjects were enrolled in this section of the study. The data collected from subjects included family history for CHD (8), family and personal history of periodontitis (9), diabetes, oral hygiene habits, and smoking history. Height and weight were also obtained from subjects for determination of body mass index.

CCHD subjects were patients with clinical and/or coronary angiography diagnosis of this disease, without unprovoked chest pain, present in the hospital setting during the final stages of recovery from a myocardial infarction, but no longer in need of intensive care or angina medication. Another source for this group were patients admitted for an upcoming surgical treatment of affected coronaries, which involved either angioplasty or bypass surgery.

ACHD subjects were patients within a recent episode of myocardial infarction, but unlike the CCHD patients, ACHD patients were under intensive care and were examined 72–96 h after hospital admission due to a heart attack. All subjects from this group were medicated against the infarction and were without chest pain or any other form of discomfort that would prevent them from having a periodontal examination.

CG subjects were recalled based on their coronary angiography, which had been performed previously to diagnose non-ischemic heart diseases, such as heart valve malfunctions or congestive heart disease. Subjects from this group must have exhibited clear coronaries without atheroma lesions and were not considered patients with CHD by the hospital's cardiologists. Only image exams from the six months prior to data collection for this study were used.

Sample size determination—was conducted with an expected SPD of 2.4 mm in the most affected group and 2.0 mm in the least affected group, with a standard deviation of 0.7 mm in each group. These expected SPD's resulted in a necessary sample size of 43 subjects per group with 80% power to detect a statistical difference at *α* = 0.05 level of significance in SPD.

Coronary angiography evaluation—all films were reanalyzed for this study regarding the extent and severity of CHD, which was determined from the following variables: number of vessels with atheroma lesions, number of atheroma lesions, lesion location, percentage of stenosis, and lesion stability. All evaluations were conducted by one experienced examiner, following established standard criteria for diagnosis (10, 11).

Periodontal evaluation—all exams were performed bedside with proper lighting and magnification for clear visualization. Each subject underwent a full-mouth examination (12, 13), which was performed by one calibrated examiner with the use of an insertion-controlled periodontal probe (HAWE Click Probe—NEOS DENTAL, Switzerland). This probe removes the possibility of tissue damage during probing with consequent creation of false clinical data that could be correlated to periodontal diseases, by the use of an articulation that clicks upon tissue resistance. The following variables were included: (a) number of teeth; (b) probing depth (PD), summarized in two variables: (i) percentage of putative active sites with active periodontitis [PD > 3 mm with bleeding on probing (BOP)], and (ii) mean PD; (c) clinical attachment level (CAL), which measures the attachment loss or gain around each tooth. CAL was stratified into three categories according to the loss of attachment, reflecting the cumulative value of the yearly rate of loss of periodontal attachment (14): (i) mild (≤3 mm CAL), (ii) moderate (4-to-6 mm CAL), and (iii) advanced (≥7 mm CAL); and (d) BOP, classified as positive or negative (15). All parameters were obtained with the examination of six sites around each present tooth. The last obtained periodontal parameter was (e) the dental plaque index (16), measured for each free tooth surface, as an indicator of oral hygiene. After data collection, subjects were classified according to the staging of periodontitis (17, 18). To facilitate comparison of subjects with advanced periodontitis to subjects with less severe periodontitis, two groups were created: one with subjects with stages one and two, and the other group with stages three and four. Subjects without periodontitis were also grouped together with subjects without teeth where tooth loss was reported to not have been caused by periodontitis forming the non-applicable (N/A) staging.

Evaluation of systemic markers—blood samples were collected from most of the subjects that underwent complete periodontal examinations, before this exam. The following variables were obtained: white cell count (CELL-DYN 3700, GMI, Ramsey, MN, USA), fibrinogen levels (coagulometric test—Sigma-Aldrich, Saint Louis, MO, USA), C-reactive protein (Sigma-Aldrich, Saint Louis, MO, USA), and serum lipid profile (dry chemistry—VITROS—Ortho Clinical Diagnostics, Raritan, NJ, USA). Blood collections were performed in the morning, with analysis following the National Institute of Cardiology's routine flow of laboratorial analysis.

Biostatistics—two analyses were performed: the first tested the correlations of periodontal parameters, coronary angiography data, epidemiologic data, and data from blood samples with the clinical CHD status of all subjects.

The second analysis tested the correlations of periodontal parameters, epidemiologic data, and data from blood samples with the number of atheroma lesions present per subject. This analysis only comprised subjects with an available coronary angiography with 93 subjects meeting this criterion.

The following tests were performed: ANOVA (with Bonferroni post-hoc analysis), Kruskal Wallis nonparametric ANOVA, Spearman's non-parametric and Pearson correlations, Student's independent samples *t*-tests, Mann-Whitney U tests, chi-square tests, and odds ratios from multiple logistic regression analysis. Tests were performed using Microsoft Excel (Microsoft Corporation, Redmond, WA, USA), Graphpad 8 (GraphPad Software, La Jolla, California, USA), and SPSS 28 (IBM, Armonk, New York, USA) softwares.

Three authors (MM, BT and MN) had complete access to all databases and are responsible for the analysis and integrity of the data.

Microbiology—additional participants were recruited, following the same exclusion criteria with the addition of completely edentulous subjects. Upon informed consent, subjects agreed to donate samples. The thirty subjects selected in this manner needed either a coronary or a carotid endarterectomy. One day before the endarterectomy, the subgingival biofilm was collected after the crown surfaces were cleaned with the help of sterile gauze. Subgingival biofilm samples were collected pooling a sample with the use of sterile Gracey curettes (Trinity, São Paulo, SP, Brazil). This sample was placed into a sterile eppendorf with 200 ml of TE buffer (tris-HCl 10 mM; EDTA 1 mM; pH 7.8, Sigma-Aldrich, Saint Louis, MO, USA). Atheroma lesions were collected immediately after surgical removal, placed in a sterile vial with sterile saline (NaCl 0.9%, transported under refrigeration and stored at −20°C until processing. The polymerase chain reaction (PCR) was performed at the laboratory facilities of the Federal University of Rio de Janeiro.

DNA extraction from biofilms followed the protocol by Nunes et al. (19). Supernatants were aliquoted in triplicates and stored at −20°C. Atheromas were sterile macerated, incubated 2 h at 55°C into 400 µl of a digestion buffer (0,25 µl/ml proteinase K; NaCl 0.1 M; tris-HCL 10 mM; EDTA 25 nM; SDS0.5%, Sigma-Aldrich, Saint Louis, MO, USA) and warmed up to 95°C for 10 min. DNA extraction continued twice with equal volumes of phenol-chloroform (tris-HCl 0.1 M, pH 8.0, saturated solution, Sigma-Aldrich, Saint Louis, MO, USA), completed by adding 50 µg of glycogen with final extraction using the same volume of chloroform. After five minutes of centrifugation to remove debris, the DNA was precipitated by the addition of two volumes of 95% ethanol and stored overnight at −20°C. Tubes were centrifuged for 10 min at 4°C. Pellets were washed with 1 ml of 70% ethanol and dissolved in 50 ml of sterile bi-distilled water, incubated at 37°C for 15 min, and then stored at −20°C.

Polymerase chain reaction (PCR)—the methodology established by Ashimoto et al. (20) was followed (Supplementary Table S1). PCR products were separated in a 1.5% agarose gel (Gibco, Thermo Fischer, Waltham, MA, USA), dissolved in a 1× TBE buffer (tris 89 mM; Boric acid 89 mM; EDTA 2.5 M, pH 8.0). To each sample was added 1/6 (v/v) of a sample buffer (50% glycerol; 10 mM EDTA; 0.25% ABF). Electrophoresis was performed under 50 Volts for 3 h. The gel was stained by an ethidium bromide solution (0.5 µg/ml) for one hour and photographed under a UV transilluminator.

## 3 Results

Demographics, blood markers and coronarography data of all participants, based on the coronary heart disease diagnosis (CHD) are presented in Table 1. CHD groups had a composition of male subjects higher than 60%, contrasting with the 48% of male subjects from the no CHD group (CG), although not statistically significant. No significant differences were observed regarding age or body mass index (BMI), with all groups having a similar prevalence of overweight. Prevalence of hypertension was also statistically similar among groups. Chronic CHD (CCHD) subjects presented with a significantly lower prevalence of positive familial history for CHD compared to acute CHD (ACHD) or the no CHD group (CG). Both CHD groups had a significantly higher prevalence of diabetes mellitus (both insulin-dependent and type 2 diabetes mellitus) and a significantly lower prevalence of “never smokers” compared to CG.

**Table 1:** Demographic, blood markers and coronariographic data stratified by ischemic heart disease diagnosis.

	No CHD (CG)	Chronic CHD	Acute CHD	Total	*P*
Female (%)	24 (52.2%)	22 (35.5%)	18 (34.0%)	64 (39.8%)	0.124
Age (Years ± SD)	59.37 (±9.07)	62.53 (±11.53)	63.57 (±11.67)	61.96 (±10.90)	0.143
Average Body Mass Index (±SD)	25.51 (±3.54)	25.92 (±3.73)	26.12 (±3.32)	25.88 (±3.52)	0.701
High Blood Pressure (%)	34 (73.9%)	41 (66.1%)	41 (77.4%)	116 (72.0%	0.387
Subjects with Coronary Angiography	46 (100%)	24 (38.7%)	23 (43.4%)	93 (57.8%)	N/A
Positive Family History of CHD (%)	34 (73.9%)	**31** (**50%)**	36 (67.9%)	101 (62.7%)	**0**.**025**
Diabetic Subjects (%)	4 (8.70%)	**17** (**27.4%)**	**17** (**32.1%)**	38 (23.6%)	**0**.**016**
Insulin-Dependent Subjects (%)	0 (0.0%)	2 (3**.23%)**	**10** (**18.9%)**	12 (7.45%)	**<0**.**001**
Female Hormone Replacement (%)	2 (8.33%)	2 (9.09%)	**6** (**33.3%)**	10 (15.6%)	**0**.**051**
Smoking Status:			** **		**0**.**001**
Current Smokers (%)	4 (8.70%)	**11** (**17.7%)**	5 (9.43%)	20 (12.4%)	** **
Former Smokers (%)	11 (23.9%)	**29** (**46.8%)**	**31** (**58.5%)**	71 (44.1%)	** **
Never Smokers (%)	31 (67.4%)	**22** (**35.5%)**	**17** (**32.1%)**	70 (43.5%)	** **
Positive Family History of Periodontitis (%)	5 (10.9%)	14 (22.6%)	11 (20.8%)	30 (18.6%)	0.269
Self-Reported History of Periodontitis (%)	17 (37.0%)	19 (30.6%)	24 (45.3%)	60 (37.3%	0.270
Subjects under Periodontal Treatment (%)	4 (8.70%)	6 (9.68%)	5 (9.43%)	15 (9.32%)	0.984
Leukocytes (±SD)	5646.7 ± 1925.5	**7643.1 ** **±** ** 2657.8**	**7381.1 ** **±** ** 2498.3**	6944.6 ± 2524.3	**0**.**002**
Monocytes (±SD)	399.3 ± 153.9	**602.7 ** **±** ** 208.4**	**650.4 ** **±** ** 275.1**	559.8 ± 245.7	**<0**.**001**
Fibrinogen (±SD)	281.9 ± 125.5	**519.5 ** **±** ** 183.4**	343.3 ± 118.4	383.7 ± 174.2	**<0**.**001**
HDL Cholesterol (±SD)	**48.6 ** **±** ** 12.4**	32.1 ± 9.20	36.2 ± 4.34	38.6 ± 11.2	**<0**.**001**
LDL Cholesterol (±SD)	**120.3 ** **±** ** 36.4**	110.8 ± 33.5	92.8 ± 40.2	107.1 ± 38.3	**0**.**011**
Triglycerides (±SD)	133.3 ± 72.1	158.0 ± 53.5	156.6 ± 74.3	150.0 ± 67.8	0.273
C-Reactive Protein (±SD)	0.47 ± 0.46	**1.75 ** **±** ** 1.88**	**3.18 ** **±** ** 3.63**	1.81 ± 2.61	**<0**.**001**
Average No. of Affected Coronaries (±SD)	–	2.58 ± 0.65	2.57 ± 0.59	2.57 ± 0.62	0.921
Average No. of Atheroma (±SD)	–	5.96 ± 3.99	5.65 ± 3.17	5.77 ± 3.58	0.711
Average No. of Atheroma > 70% Stenosis (±SD)	–	2.38 ± 2.00	2.83 ± 2.42	2.60 ± 2.20	0.489
Average No. of Unstable Atheroma	–	0.42 ± 0.58	**0.87 ** **±** ** 0.81**	0.64 ± 0.74	**0**.**033**

CHD, coronary heart disease; CG, control group; SD, standard deviation; HDL, high-density lipoprotein; LDL, low-density lipoprotein.

Bold values denotes statistical difference between groups.

Inflammatory markers were elevated among CHD subjects, as presented in Table 1. Leukocytes and monocytes were significantly elevated among subjects in both CHD groups (CCHD and ACHD) compared to the no CHD group (CG). Fibrinogen levels were significantly higher among CCHD subjects compared to both ACHD subjects and CG subjects. C-reactive protein (CRP) was also significantly elevated in both CHD groups compared to the no CHD group (CG), with the acute CHD group (ACHD) exhibiting significantly higher C-reactive protein compared to C-reactive protein in the chronic CHD group (CCHD).

The lipid profile also presented statistically significant differences among groups. Low-density lipoprotein (LDL) and high-density lipoprotein (HDL) were significantly higher among CG subjects compared to both CHD groups. CCHD Subjects had even lower HDL levels compared to ACHD (*p *= 0.027).

There were no statistically significant differences in the number of affected coronaries between groups with CHD, also the number of atheroma lesions (including those with more than 70% of stenosis) was similar. Reflecting the clinical cardiological diagnosis, ACHD presented more unstable atheroma lesions compared to CCHD (Table 1).

**Figure 1:**
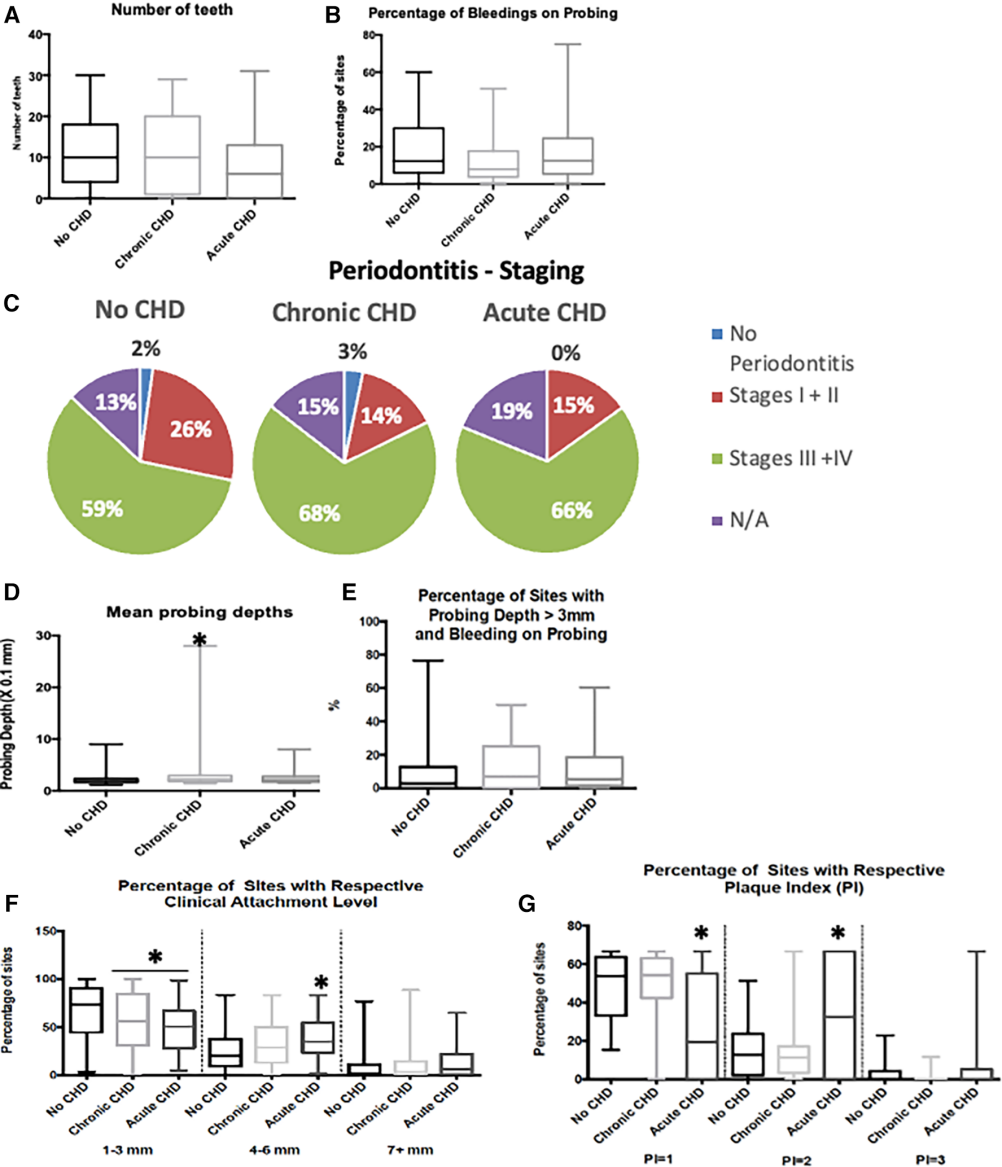
Oral and periodontal data sorted by CHD diagnosis. CHD, coronary heart disease; N/A, non-applicable; mm, millimetres. “*” denotes statistical difference when compared to the “No CHD” group. (**A**) – number of teeth per group, (**B**) – sites with gingival bleeding during examination, (**C**–**F**) – diagnostic data on periodontitis, based on its staging (**C**), mean probing depth from the clinical exam (**D**), clinical evidence of activity (**E**), tissue damage (**F**). (**G**) – oral hygiene status

No statistically significant difference in the number of teeth was detected among the groups (Figure 1A, *p *= 0.124). Similarly, no statistically significant difference was obtained for the percentage of sites with bleeding on probing (BOP) among the three groups (Figure 1B, *p *= 0.125). Periodontal staging was similar among the three groups (Figure 1C, *p *= 0.380), and percentage of sites with putative clinical active periodontitis was also similar among the three groups (Figure 1E, *p *= 0.489).

CCHD subjects had significantly greater mean probing depths (PD) compared to the no CHD group (CG) (Figure 1D, *p *= 0.022, Mann–Whitney *U* test comparing PD for CCHD to PD for CG). Loss of periodontal attachment, as indicated by clinical attachment level (CAL), demonstrated statistically significant differences for mean CAL among the three groups (CG, CCHD, ACHD) (*p *= 0.007, Kruskal-Wallis nonparametric ANOVA). Comparisons were made among the CHD groups according to the percentage of CAL sites in the following categories: (1) ≤3 mm CAL (mild loss), (2) 4-to-6 mm CAL (moderate loss), (3) ≥7 mm CAL (severe loss). CG demonstrated a higher percentage of sites with mild attachment loss (≤3 mm CAL) compared to both CCHD and ACHD groups (Figure 1F, *p *= 0.005, Kruskal-Wallis nonparametric ANOVA). Percentage of sites with moderate loss (4-to-6 mm CAL) varied significantly across CHD groups (*p *< 0.001, Kruskal-Wallis nonparametric ANOVA) with the CCHD group demonstrating a greater percentage of sites with moderate loss (4-to-6 mm CAL) compared to CG (*p *< 0.001, Mann-Whitney U test comparing percentage of sites with moderate loss for CCHD group to percentage of sites with moderate loss for CG). The difference in percentage of sites with moderate loss (4-to-6 mm CAL) between the ACHD group and CG failed to achieve statistical significance (*p *= 0.071, Mann–Whitney *U* test comparing percentage of sites with moderate loss for ACHD group to percentage of sites with moderate loss for CG). Percentage of sites with severe loss (≥7 mm CAL) did not vary significantly across CHD groups (*p *= 0.100, Kruskal-Wallis nonparametric ANOVA).

Subjects with ACHD and under intensive care had worse mean dental plaque index compared to the mean dental plaque indices of the CCHD group and CG (*p *< 0.001, ANOVA and Bonferroni-corrected multiple comparisons for mean plaque index of ACHD group compared to mean plaque index of CCHD group and for mean plaque index of ACHD group compared to mean plaque index for CG). The ACHD group had a lower percent of sites with low levels of dental plaque (plaque index of 1) (Figure 1G, *p *< 0.001, ANOVA and Bonferroni-corrected multiple comparisons for % of sites with plaque index of 1 for ACHD group compared to % of sites with plaque index of 1 for CCHD group and for % of sites with plaque index of 1 for ACHD group compared to % of sites with plaque index of 1 for CG) and a higher percent of sites with moderate plaque levels (% of sites with plaque index of 2) compared to the CCHD group and CG (Figure 1G, *p *< 0.001, ANOVA and Bonferroni-corrected multiple comparisons for % of sites with plaque index of 2 for ACHD group compared to % of sites with plaque index of 2 for CCHD group and for % of sites with plaque index of 2 for ACHD group compared to % of sites with plaque index of 2 for CG), indicating deficient oral hygiene.

Multiple logistic regression of significant systemic and periodontal variables was fit comparing all subjects with CHD (ACHD or CCHD) to CG. The final parsimonious model shown provides information on those factors that may favor the onset of CHD via odds ratios (Table 2). Being a current smoker increased the odds of CHD by almost 4.4 times when compared to non-smokers. Former smokers had increased risk of CHD of 3.8 times when compared to non-smokers. Subjects with a mean CAL greater than 2.7 mm had 3.8 times the likelihood of CHD compared to subjects with mean CAL less than 2.7 mm. Subjects with 25% or more sites with moderate CAL (4-to-6 mm) were nearly 3.2 times more likely to have CHD compared to subjects with a lower percent of sites with moderate CAL (4-to-6 mm).

**Table 2:** Significative base logistic regression model for all CHD (ACHD or CCHD) versus healthy (CG).

Variable	*p*	Odds ratio	95% C.I. for Odds ratio	
			Lower	Upper
Age (in years)	0.019	1.04	1.01	1.08
Former Smoker vs. Nonsmoker	0.036	3.82	1.09	13.41
Current Smoker vs. Nonsmoker	0.001	4.38	1.84	10.43
Average CAL of 2.7 mm or higher	0.016	3.83	1.28	11.45
Subjects with ≥25% CAL of 4–6 mm	0.037	3.19	1.07	9.45

CAL, clinical atachment level/loss.

Data were further analyzed with only subjects with coronary angiographic exams recategorized into groups based upon the number of detected atheromas. Subjects without CHD (healthy control group) were relabeled as the group with clean coronaries (zero atheromas), with 46 subjects. 47 subjects with CHD and coronary angiography were split in half: the first half comprised of 23 subjects with 1-to-4 atheromas, the second half composed of 24 subjects with 5-to-18 atheromas.

Initial demographic and blood collection data stratified by atheroma group (0, 1–4, 5–18) are shown in Table 3. The group with 5-to-18 atheroma lesions was almost completely composed of male subjects with 87.5% (21/24) of that group consisting of men, the group with 1-to-4 atheroma lesions had almost 61% (14/23) who were men. No significant differences in age, BMI, or hypertension were observed among the groups classified by number of atheroma lesions. Patients with more atheroma lesions had a higher prevalence of diabetes mellitus (*p *= 0.004), more women under hormone replacement therapy (*p *= 0.033), and a greater prevalence of current or former smokers (*p *= 0.014) compared to the group without atheroma lesions.

**Table 3:** Demographic and systemic blood data stratified by number of atheroma.

Number of Atheroma	0	1–4	5–18	Total	*p*
Female (%)	24 (52.2%)	9 (39.1%)	**3** (**12.5%)**	36 (38.7%)	**0**.**005**
Age—in years (± SD)	59.4 (±9.07)	62.6 (±11.0)	61.2 (±10.8)	60.6 (±9.99)	0.431
Average Body Mass Index (±SD)	25.5 (±3.54)	26.6 (±3.09)	25.6 (±2.36)	25.8 (±3.15)	0.381
High Blood Pressure (%)	34 (73.9%)	18 (78.3%)	20 (83.3%)	72 (77.4%)	0.666
Positive Family History of CHD (%)	34 (73.9%)	17 (73.9%)	14 (58.3%)	65 (69.9%)	0.358
Diabetes Mellitus (%)	4 (8.7%)	**9** (**39.1%)**	**9** (**37.5%)**	22 (23.7%)	**0**.**004**
Insulin Dependent Diabetes Mellitus (%)	0 (0.0%)	**2** (**8.7%)**	**4** (**16.7%)**	6 (6.5%)	**0**.**023**
Women under Hormone Replacement (%)	2 (8.3%)	**2** (**22.2%)**	**2** (**66.7%)**	6 (16.7%)	**0**.**033**
Smoking Status:		** **	** **		**0**.**014**
Current Smokers (%)	4 (8.7%)	**3** (**13.0%)**	**3** (**12.5%)**	10 (10.8%)	** **
Former Smokers (%)	11 (23.9%)	**10** (**43.5%)**	**15** (**62.5%)**	36 (38.7%)	
Never Smokers (%)	**31** (**67.4%)**	10 (43.5%)	6 (25.0%)	47 (50.5%)	
Self-reported History of Periodontitis	17 (37.0%)	4 (17.4%)	12 (50.0%)	33 (35.5%)	0.063
Family History of Periodontitis (%)	5 (10.9%)	**11** (**47.8%)**	4 (16.7%)	20 (21.5%)	**0**.**002**
Subjects Under Periodontal Treatment (%)	4 (8.7%)	2 (8.7%)	2 (8.3%)	8 (8.6%)	0.999
Leukocytes (±SD)	5646.7 ± 1925.5	**7596.0 ** **±** ** 3219.4**	**7829.5 ** **±** ** 2101.2**	6751.6 ± 2524.4	**0**.**003**
Monocytes (±SD)	399.3 ± 153.9	**697.3 ** **±** ** 308.8**	**617.9 ** **±** ** 206.5**	534.0 ± 247.8	**<0**.**001**
Fibrinogen (±SD)	281.9 ± 125.5	**445.5 ** **±** ** 186.0**	**425.8 ** **±** ** 215.7**	365.6 ± 185.9	**0**.**004**
Triglycerides (±SD)	133.3 ± 72.1	163.7 ± 63.0	178.7 **±** 62.6	153.9 ± 69.3	0.066
HDL Cholesterol (±SD)	48.6 ± 12.4	**38.2 ** **±** ** 9.3**	**32.8 ** **±** ** 5.05**	41.5 ± 12.1	**<0**.**001**
LDL Cholesterol (±SD)	120.3 ± 36.4	96.1 ± 35.7	112.7 ± 36.7	112.4 ± 37.0	0.119
C-Reactive Protein (±SD)	0.47 ± 0.46	**2.81 ** **±** ** 4.40**	**2.76–2.81**	1.63 ± 2.82	**0**.**005**

SD, standard deviation; HDL, high-density lipoprotein; LDL, low-density lipoprotein.

Bold values denotes statistical difference between groups.

Blood markers were grouped by number of atheroma lesions (no lesions, 1 to 4 lesions, 5 to 18 lesions) with results shown in Table 3. Patients with atheroma lesions had significantly higher values for leukocytes, monocytes, fibrinogen, and C-reactive protein compared to subjects without atheroma lesions. Lipoprotein analysis revealed lower HDL levels among subjects with atheroma lesions when compared to subjects with clean coronaries. When the two atheroma-lesion groups were compared, HDL was lower among subjects with 5-to-18 lesions compared to subjects with 1-to-4 atheroma lesions (*p* = 0.037).

**Figure 2:**
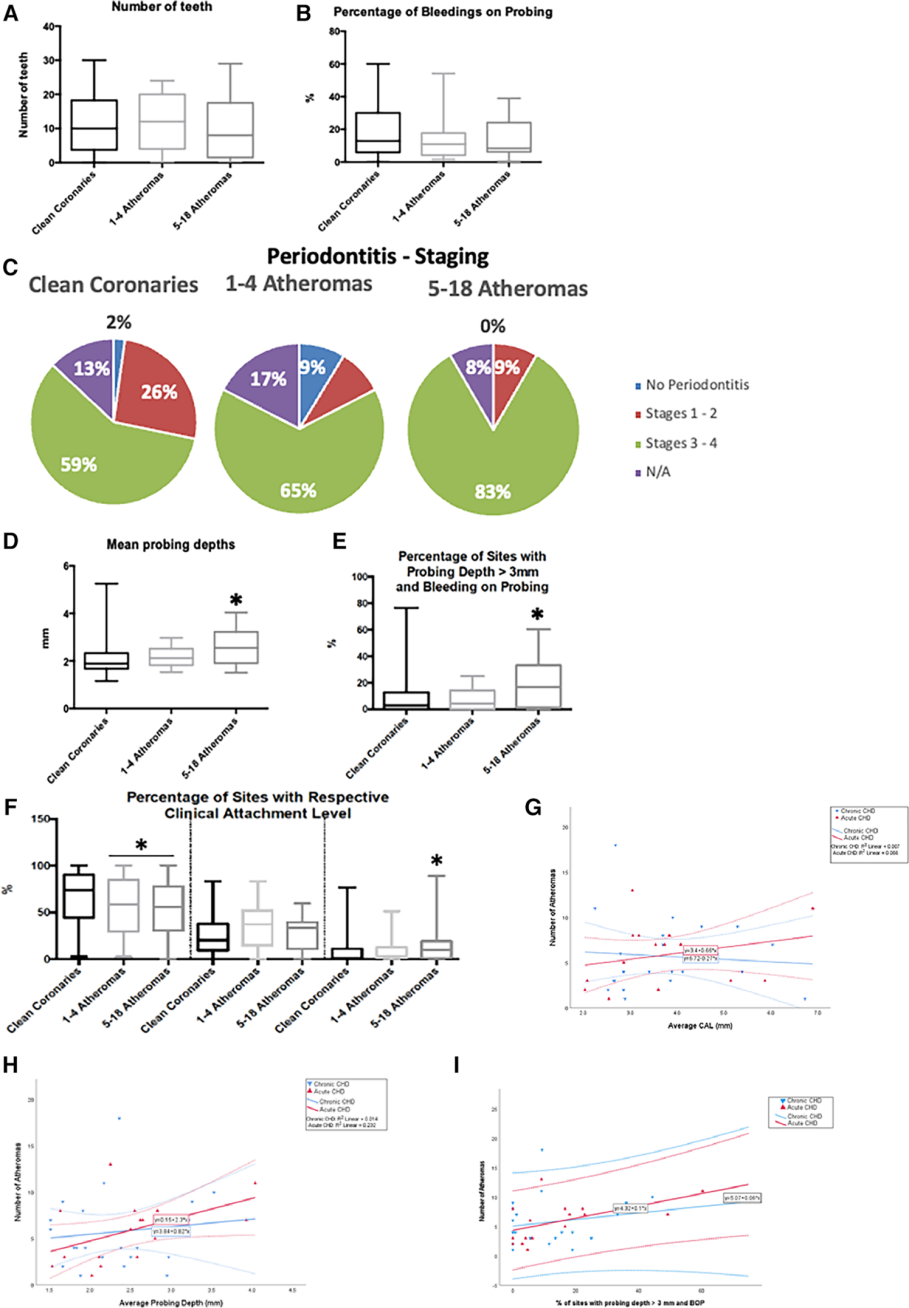
Oral and periodontal data sorted by the number of atheromas. N/A, non-applicable; mm, millimetres. “*” denotes statistical difference when compared to the “No CHD” group. (**A**) – number of teeth per group, (**B**) – sites with gingival bleeding during examination, (**C**–**F**) – diagnostic data on periodontitis, based on its staging (**C**), mean probing depth from the clinical exam (**D**), clinical evidence of activity (**E**), tissue damage (**F**). (**G**–**I**) – Linear correlations divided by the coronary heart disease status to the number of atheromas, based on periodontal tissue damage (**G**), mean probing depth from the clinical exam (**H**), and clinical evidence of activity (**I**).

Atheroma quantification analysis did not show any differences in the number of teeth by atheroma lesion group (Figure 2A, *p *= 0.739) or in percent sites with bleeding on probing by atheroma lesion group (Figure 2B, *p *= 0.620). Staging of periodontitis across atheroma groups failed to achieve statistical significance (Figure 2C, *p *= 0.072). However, the 5-to-18 atheroma-lesion group had greater PD (Figure 2D, *p* = 0.016) and a higher percent of active sites (*p* = 0.012, Figure 2E). The group with 5–18 atheroma lesions demonstrated more advanced CAL when compared to the other groups, with a higher percent of sites with severe CAL (≥7 mm CAL) (*p *= 0.032, Mann–Whitney *U* test comparing percent sites with severe CAL in 5-to-18 atheroma-lesion group compared to 0-to-4 atheroma-lesion group, Figure 2F). In contrast, a greater percent of sites with mild CAL (≤3 mm CAL) was more prevalent in the group without atheroma lesions when compared to both atheroma groups (1-to-4 lesions, 5-to-18 lesions) (*p *= 0.004, Mann–Whitney *U* test comparing 64.5% sites with mild CAL in group without atheroma lesions to 47.9% sites with mild CAL in group with 1-to-18 atheroma lesions).

Correlations showed that CAL is associated with more atheroma lesions in the ACHD group (Figure 2G), while mean PD and percent active periodontitis sites were associated with more atheroma lesions among all subjects with CHD (ACHD and CCHD) (Figures 2H,I).

Table 4 presents independent periodontal risk factors for CHD-related outcomes. A greater number of sites with active periodontitis was a risk factor for a greater number of atheroma lesions and the formation of larger atheroma lesions. Increasing percent of sites with severe CAL (≥7 mm CAL) was also associated with an increase in atheroma lesion size. Greater percents of moderate or severe CAL were associated with increased numbers of monocytes, inflammatory markers, like CRP and fibrinogen, and a dyslipidemia profile, with high triglycerides and low HDL levels. In contrast, mild CAL indicated less systemic inflammation, with lower monocyte, CRP, and effect on the lipid profile, with higher HDL levels. BOP was also characterized as an independent risk factor for systemic inflammation, with higher monocyte and CRP values, as with lower levels of HDL.

**Table 4:** Spearman's correlations.

Outcome	Variable (sig.)
More arteries with atheroma	↑ %Sites with PD > 3 mm + BOP (0.04)
More atheroma	↑ %Sites with PD > 3 mm + BOP (0.05)
Larger atheroma (>70% Stenosis)	↑ %Sites with PD > 3 mm + BOP (0.00); ↑ Average PD (0.00)
Higher Monocytes counts	↓ CAL 3 mm or Less (0.03); ↑ CAL 7 + mm (0.04); ↑ CAL 7 + mm (0.04); ↑ BOP (0.04)
Higher Fibrinogen Levels	↑ CAL 4–6 mm (0.01)
Higher Triglycerides Levels	↑ Average PD (0.03); ↑ CAL 4–6 mm (0.02)
Lower HDL Levels	↓ CAL 3 mm or Less (0.01); ↑ CAL 4–6 mm (0.00); ↑ CAL 7 + mm (0.03); ↑ BOP (0.00)
Higher C-Reactive Protein Levels	↓ CAL 3 mm or Less (0.01); ↑ CAL 7 + mm (0.03); ↑ CAL 4–6 mm (0.02); ↑ BOP (0.01)

HDL, high-density lipoprotein; PD, probing depth; BOP, bleeding on probing; CAL, clinical attachment level/loss; ↑, agonistic effect; ↓, antagonistic effect.

The microbiologic analysis of atheroma is presented, with demographics from all thirty subjects/atheroma shown in Supplementary Table S2. Since coronary bypass associated with endarterectomy is commonly replaced by the less-invasive angioplasty, atheroma lesions from carotid arteries were included in this analysis since they are still performed to prevent strokes.

**Figure 3:**
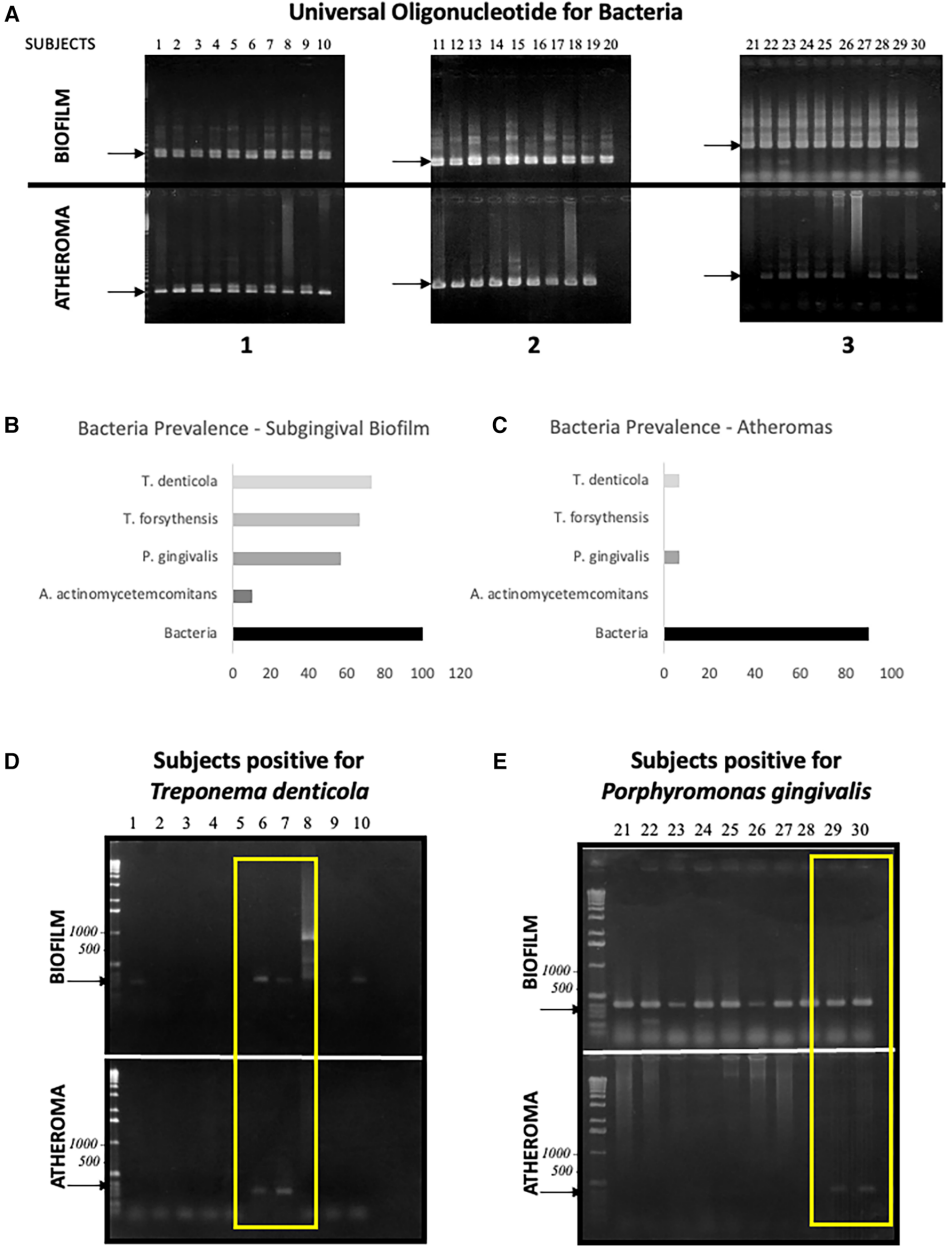
Polymerase chain-reaction for periodontal bacteria. (**A**–**C**) – universal bacterial nucleotide for the 16s hypervariant region's DNA: (**A**) signals from oral biofilm and atheroma samples, (**B**) biofilm prevalence for the universal 16s nucleotide and tested bacteria, (**C**) atheroma prevalence for the universal 16s nucleotide and tested bacteria. (**D**–**E**) – signals of co-colonization of atheroma and subgingival plaque by tested bacteria (yellow inserts).

Bacteria were found in all subgingival biofilm samples (Figures 3A,B). For the four bacteria analyzed, a prevalence higher than 50% among biofilm samples was found for all the bacteria except for *Aggregatibacter actnomycetemcomitans* (Aa), which demonstrated a prevalence of 10% (Figure 3B).

Although bacteria were detected in the atheromas with a prevalence of 90% (Figures 3A,C), only two of the four pathogens targeted were detected in lesions: *Treponema denticola* and *Porphyromonas gingivalis*. Both with a low (6.66%) prevalence (Figures 3C–E). The four patients with positive results for the targeted bacteria also presented positive results for the same species in the subgingival biofilm (Figures 3D,E—delimited by the yellow rectangles). From 22 subjects infected with *Treponema denticola* in the subgingival biofilm, 2 presented this species inside the atheroma with 9.09% rate of mutual infection. In the same manner, *Porphyromonas gingivalis* possesses an 11.76% mutual infection rate, found in the subgingival biofilm of 17 subjects, but only for 2 atheroma lesions.

## 4 Discussion

Not every classic risk factor for coronary heart disease (21) (CHD) was associated with it in this cross-sectional analysis of a population from the greater Rio de Janeiro area. Hypertension, elevated body-mass index (BMI), higher low-density lipoprotein (LDL), and triglycerides were not related to the clinical diagnosis of CHD nor the existence or increase in the number of coronary atheroma lesions. Diabetes and smoking, which are common risk (and confounding) factors for CHD and periodontitis (22, 23), were associated with CHD, as were the presence and the increased amount of atheroma lesions. However, multiple logistic regression failed to show diabetes as a significant CHD risk factor, although smoking increased the risk of CHD by 4.2 times and 3.8 times for current smokers and former smokers, respectively.

The diagnosis of CHD and an increased number of atheroma lesions were directly associated with higher levels of all systemic inflammatory markers and were inversely associated to high-density lipoprotein (HDL) levels, a protective factor for CHD and atheroma formation (24). As CHD is considered an inflammatory disease (6), elevation of inflammatory markers is expected in response to risk factors, such as positive CHD family history, diabetes, and smoking. However, as Spearman's correlation demonstrated, clinical signs of periodontal disease, such as advanced clinical attachment loss (CAL) or bleeding on probing (BOP), were also associated with this systemic inflammation/low HDL profile.

Regarding CAL, it is important to note that even an overall moderate loss increased the risk for CHD around four times, comparable to the impact of smoking, and subjects with more than 25% of sites with moderate-to-advanced CAL had three-times higher CHD risk, attributing a risk potential for periodontitis comparable to classic CHD risk factors. Previous studies demonstrated that inflammatory markers, such as C-reactive protein, fibrinogen, interleukin-6, triglycerides, and LDL, were reduced following the treatment of periodontitis, which yields evidence of the impact of this disease on systemic inflammation (25, 26), including a study based on individuals from the same institution as those presented in this manuscript (27).

Based on all risk indicators and associations presented so far, one challenge is to establish a temporal relationship between these two diseases, which is yet to be proven. Alternatively, we sought to determine if the severity of periodontitis could be somehow correlated to the risk and burden of coronary injury, which leads to atheroma formation.

When analyzing the periodontal status of the groups sorted by coronary clinical diagnosis or the number of atheroma lesions, some differences are noted. Mild CAL is associated with the CG, being associated with both the clinical absence of CHD and with clean coronaries (i.e., no atheroma lesions). Since CAL is an historic signal for the progression of periodontitis (i.e., this disease burden for periodontitis), the data demonstrate attenuated destruction from the inflammation of the periodontium in the same subjects without CHD/atheroma. Conversely, subjects with more atheroma lesions are those with more sites of advanced CAL, in which the CAL levels are even compatible with poor tooth prognosis (28). This case-control analysis points out how advanced levels of periodontal injury, via increased CAL, are found with equivalent injury among coronary arteries, via a greater number of atheroma lesions and increased size of atheroma lesions. However, this association only presented as a linear correlation among subjects with acute CHD (ACHD).

Surprisingly, data demonstrated an association between active periodontitis and an increased number of atheroma lesions. Subjects with 5-to-18 atheroma lesions presented with higher mean probing depths (PD) and a greater percent of active periodontitis sites with both yielding a linear correlation to the number of atheroma lesions for chronic CHD (CCHD) and acute CHD (ACHD). Spearman's correlation reinforces that active periodontitis is associated with an increased number of atheroma lesions and an increased size of atheroma lesions. This provides some evidence that periodontitis is associated with atheroma lesions, which may be the result of the impact of periodontitis on systemic inflammation and/or bacteremia.

Although the data analyzed were from a cross-sectional data collection, the chronic pattern of progression of periodontitis (29, 30) leads to the conclusion that the moment of activity detected during this study was not a standalone event, with high probability of cumulative recurrence in most, if not all, subjects in years before the periodontal exam for this study was conducted.

ACHD subjects, under intensive care, presented poorer oral hygiene based on the plaque index (16), providing relevance to oral hygiene procedures to avoid post-surgical or post-ICU complications, such as nosocomial pneumonia (31), and increase in bacteremia. The brief microbiologic analysis of this study detected pathogens with established relationships with periodontitis in the subgingival biofilm of the sampled population (32). Hence, a putative etiologic factor for periodontitis existed in more than 80% of the participants that donated atheroma lesions. However, only four of thirty atheroma lesions have one of these pathogens, also detected in the subgingival biofilm of the same individuals. Atheroma lesions presented prevalence in the 90% range for bacterial DNA, possibly from not-known or not-tested periodontal pathogens, commensal oral, or even extra-oral bacteria. Current microbiome sequencing techniques could assist in mapping the sources of bacteria found in atheroma lesions to a wider extent of what was conducted by us in this preliminary assay. It is safe to state that, although finding the bacterial DNA in atheroma lesions is common (33–38), the evidence for their pathogenic role in the formation and development of atheroma lesions needs confirmation, due to the heterogeneity of microbiological methods and results.

The study of the association between periodontal diseases and CHD has been a challenge for the last three decades (1). Robust epidemiologic data and venues of biologic plausibility exist (5). Both diseases co-exist and could exert mutual influence in some individuals (39, 40). Within the scope of this study, similarities in the pathologic burden from periodontitis and coronary atheroma lesions were compatible to the point of the possible existence of some proportionality between them with findings that reflect and expand results from a similar study (7). Finally, given the expense and invasive nature of advanced cardiologic diagnostic procedures, the associations observed in this study suggest that an inexpensive periodontal examination and diagnosis, combined with common inflammatory and lipidic blood markers, has shown some preliminary capability to be used as an alternative to predict the onset and risk for coronary atheroma lesions (and thus, CHD), as these lesions not only have similarities in their numbers and rate of progression, but atheroma formation would be directly associated with active periodontitis. This alternative approach could be particularly useful in underserved populations where advanced diagnostic cardiologic procedures are often not available. Larger cohorts and reproduction of this study in different populations will be necessary to confirm the feasibility of what we suggest.

## Data Availability

The raw data supporting the conclusions of this article will be made available by the authors, without undue reservation.

## References

[1] Beck JD Papapanou PN Philips KH Offenbacher S. Periodontal medicine: 100 years of progress. J Dent Res. (2019) 98:1053–62. 10.1177/0022034519846113 31429666

[2] Monsarrat P Blaizot A Kemoun P Ravaud P Nabet C Sixou M Clinical research activity in periodontal medicine: a systematic mapping of trial registers. J Clin Periodontol. (2016) 43:390–400. 10.1111/jcpe.12534 26881700

[3] Friedewald VE Kornman KS Beck JD Genco R Goldfine A Libby P The American journal of cardiology and journal of periodontology Editors’ consensus: periodontitis and atherosclerotic cardiovascular disease. Am J Cardiol. (2009) 104:59–68. 10.1016/j.amjcard.2009.05.002 19576322

[4] Sanz M Del Castillo AM Jepsen S Gonzalez-Juanatey JR D'Aiuto F Bouchard P Periodontitis and cardiovascular diseases. Consensus report. Glob Heart. (2020) 15:1. 10.5334/gh.400 32489774 PMC7218770

[5] Loesche WJ Lopatin DE. Interactions between periodontal disease, medical diseases and immunity in the older individual. Periodontol 2000. (1998) 16:80–105. 10.1111/j.1600-0757.1998.tb00117.x 10337306

[6] Ross R. Atherosclerosis–an inflammatory disease. N Engl J Med. (1999) 340:115–26. 10.1056/NEJM199901143400207 9887164

[7] Amabile N Susini G Pettenati-Soubayroux I Bonello L Gil JM Arques S Severity of periodontal disease correlates to inflammatory systemic status and independently predicts the presence and angiographic extent of stable coronary artery disease. J Intern Med. (2008) 263:644–52. 10.1111/j.1365-2796.2007.01916.x 18205762

[8] Crouch MA Gramling R. Family history of coronary heart disease: evidence-based applications. Prim Care. (2005) 32:995–1010. 10.1016/j.pop.2005.09.008 16326224

[9] Blicher B Joshipura K Eke P. Validation of self-reported periodontal disease: a systematic review. J Dent Res. (2005) 84:881–90. 10.1177/154405910508401003 16183785

[10] Scanlon PJ Faxon DP Audet AM Carabello B Dehmer GJ Eagle KA ACC/AHA guidelines for coronary angiography. A report of the American college of cardiology/American heart association task force on practice guidelines (committee on coronary angiography). developed in collaboration with the society for cardiac angiography and interventions. J Am Coll Cardiol. (1999) 33:1756–824. 10.1016/S0735-1097(99)00126-6 10334456

[11] Ellis SG Vandormael MG Cowley MJ DiSciascio G Deligonul U Topol EJ Coronary morphologic and clinical determinants of procedural outcome with angioplasty for multivessel coronary disease. Implications for patient selection. Multivessel angioplasty prognosis study group. Circulation. (1990) 82:1193–202. 10.1161/01.CIR.82.4.1193 2401060

[12] Armitage GC. Periodontal diseases: diagnosis. Ann Periodontol. (1996) 1:37–215. 10.1902/annals.1996.1.1.37 9118264

[13] Research S Therapy C. Position paper: diagnosis of periodontal diseases. J Periodontol. (2003) 74:1237–47. 10.1902/jop.2003.74.8.1237 29539063

[14] Needleman I Garcia R Gkranias N Kirkwood KL Kocher T Iorio AD Mean annual attachment, bone level, and tooth loss: a systematic review. J Clin Periodontol. (2018) 45(20):S112–29. 10.1002/JPER.17-0062 29926483

[15] Chaves ES Wood RC Jones AA Newbold DA Manwell MA Kornman KS. Relationship of “bleeding on probing” and “gingival index bleeding” as clinical parameters of gingival inflammation. J Clin Periodontol. (1993) 20:139–43. 10.1111/j.1600-051X.1993.tb00328.x 8436633

[16] Loe H. The gingival index, the plaque index and the retention index systems. J Periodontol. (1967) 38:610–6. 10.1902/jop.1967.38.6.610 5237684

[17] Papapanou PN Sanz M Buduneli N Dietrich T Feres M Fine DH Periodontitis: consensus report of workgroup 2 of the 2017 world workshop on the classification of periodontal and peri-implant diseases and conditions. J Clin Periodontol. (2018) 45(20):S162–70. 10.1111/jcpe.12946 29926490

[18] Tonetti MS Greenwell H Kornman KS. Staging and grading of periodontitis: framework and proposal of a new classification and case definition. J Periodontol. (2018) 89(1):S159–72. 10.1002/JPER.18-0006 29926952

[19] Biarnes J Barrientos A Ricart W Nunes V Fernandez-Castaner M Soler J. Diabetes mellitus associated with the A3243G mutation of mitochondrial DNA. Apropos a Case. Med Clin. (1999) 112:99–101. 10074618

[20] Ashimoto A Chen C Bakker I Slots J. Polymerase chain reaction detection of 8 putative periodontal pathogens in subgingival plaque of gingivitis and advanced periodontitis lesions. Oral Microbiol Immunol. (1996) 11:266–73. 10.1111/j.1399-302X.1996.tb00180.x 9002880

[21] Virani SS Alonso A Benjamin EJ Bittencourt MS Callaway CW Carson AP Heart disease and stroke statistics-2020 update: a report from the American heart association. Circulation. (2020) 141:e139–596. 10.1161/CIR.0000000000000757 31992061

[22] Hyman JJ Winn DM Reid BC. The role of cigarette smoking in the association between periodontal disease and coronary heart disease. J Periodontol. (2002) 73:988–94. 10.1902/jop.2002.73.9.988 12296599

[23] Southerland JH Taylor GW Moss K Beck JD Offenbacher S. Commonality in chronic inflammatory diseases: periodontitis, diabetes, and coronary artery disease. Periodontol 2000. (2006) 40:130–43. 10.1111/j.1600-0757.2005.00138.x 16398690

[24] Arora S Patra SK Saini R. HDL-A molecule with a multi-faceted role in coronary artery disease. Clin Chim Acta. (2016) 452:66–81. 10.1016/j.cca.2015.10.021 26519003

[25] Bokhari SA Khan AA Butt AK Azhar M Hanif M Izhar M Non-surgical periodontal therapy reduces coronary heart disease risk markers: a randomized controlled trial. J Clin Periodontol. (2012) 39:1065–74. 10.1111/j.1600-051X.2012.01942.x 22966824

[26] Caula AL Lira-Junior R Tinoco EM Fischer RG. The effect of periodontal therapy on cardiovascular risk markers: a 6-month randomized clinical trial. J Clin Periodontol. (2014) 41:875–82. 10.1111/jcpe.12290 25041550

[27] Vidal F Cordovil I Figueredo CM Fischer RG. Non-surgical periodontal treatment reduces cardiovascular risk in refractory hypertensive patients: a pilot study. J Clin Periodontol. (2013) 40:681–7. 10.1111/jcpe.12110 23639076

[28] McGuire MK Nunn ME. Prognosis versus actual outcome. III. The effectiveness of clinical parameters in accurately predicting tooth survival. J Periodontol. (1996) 67:666–74. 10.1902/jop.1996.67.7.666 8832477

[29] Loe H Anerud A Boysen H Morrison E. Natural history of periodontal disease in man. Rapid, moderate and no loss of attachment in Sri Lankan laborers 14 to 46 years of age. J Clin Periodontol. (1986) 13:431–45. 10.1111/j.1600-051X.1986.tb01487.x 3487557

[30] Loos BG Van Dyke TE. The role of inflammation and genetics in periodontal disease. Periodontol 2000. (2020) 83:26–39. 10.1111/prd.12297 32385877 PMC7319430

[31] Zuanazzi D Souto R Mattos MB Zuanazzi MR Tura BR Sansone C Prevalence of potential bacterial respiratory pathogens in the oral cavity of hospitalised individuals. Arch Oral Biol. (2010) 55:21–8. 10.1016/j.archoralbio.2009.10.005 19939349

[32] Socransky SS Haffajee AD Cugini MA Smith C Kent RL Jr. Microbial complexes in subgingival plaque. J Clin Periodontol. (1998) 25:134–44. 10.1111/j.1600-051X.1998.tb02419.x 9495612

[33] Chiu B. Multiple infections in carotid atherosclerotic plaques. Am Heart J. (1999) 138:S534–536. 10.1016/S0002-8703(99)70294-2 10539867

[34] Haraszthy VI Zambon JJ Trevisan M Zeid M Genco RJ. Identification of periodontal pathogens in atheromatous plaques. J Periodontol. (2000) 71:1554–60. 10.1902/jop.2000.71.10.1554 11063387

[35] Koren O Spor A Felin J Fak F Stombaugh J Tremaroli V Human oral, gut, and plaque microbiota in patients with atherosclerosis. Proc Natl Acad Sci U S A. (2011) 108(1):4592–8. 10.1073/pnas.1011383107 20937873 PMC3063583

[36] Lindskog Jonsson A Hallenius FF Akrami R Johansson E Wester P Arnerlov C Bacterial profile in human atherosclerotic plaques. Atherosclerosis. (2017) 263:177–83. 10.1016/j.atherosclerosis.2017.06.016 28646792

[37] Zaremba M Gorska R Suwalski P Kowalski J. Evaluation of the incidence of periodontitis-associated bacteria in the atherosclerotic plaque of coronary blood vessels. J Periodontol. (2007) 78:322–7. 10.1902/jop.2006.060081 17274722

[38] Zhang YM Zhong LJ Liang P Liu H Mu LT Ai SK. Relationship between microorganisms in coronary atheromatous plaques and periodontal pathogenic bacteria. Chin Med J. (2008) 121:1595–7. 10.1097/00029330-200808020-00018 18982875

[39] Munz M Richter GM Loos BG Jepsen S Divaris K Offenbacher S Genome-wide association meta-analysis of coronary artery disease and periodontitis reveals a novel shared risk locus. Sci Rep. (2018) 8:13678. 10.1038/s41598-018-31980-8 30209331 PMC6135769

[40] Schaefer AS Richter GM Groessner-Schreiber B Noack B Nothnagel M El Mokhtari NE Identification of a shared genetic susceptibility locus for coronary heart disease and periodontitis. PLoS Genet. (2009) 5:e1000378. 10.1371/journal.pgen.1000378 19214202 PMC2632758

