# Supplementary material for: Coronary atherosclerosis and periodontitis have similarities in their clinical presentation
Source: Front Oral Health. 2024 Jan 16;4:1324528. doi: 10.3389/froh.2023.1324528 (PMC10825671; doi:10.3389/froh.2023.1324528)
Supplement: Supplementary file 1 [file Table1.docx]

Supplementary Table1. Oligonucleotides’ (dNTPs) sequences used, position, size and GeneBank access number

| Primer | Sequence (5’- 3’) | Position and Size (pb) | GeneBank # |
| --- | --- | --- | --- |
| Universal | GAT TAG ATA CCC TGG TAG TCC AC  CCC GGG AAC GTA TTC ACC G | 786 – 1387 (602) | NC002695 |
| *Treponema denticola* | TAA TAC CGA ATG TGC TCA TTT ACA T  TCA AAG AAG CAT TCC CTC TTC TTC TTA | 193 – 508 (316) | NC002967 |
| *Tannerella forsythensis* | GCG TAT GTA ACC TGC CCG CA  TGC TTC AGT GTC AGT TAT ACC T | 120 – 760 (641) | NC003915 |
| *Porphyromonas gingivalis* | AGG CAG CTT GCC ATA CTG CG  ACT GT AGC AAC TAC CGA TGT | 729 – 1132 (404) | NC002950 |
| *Aggregatibacter actinomycetemcomitans* | ATG CCA ACT TGA CGT TAA AT  AAA CCC ATC TCT GAG TTC TTC TTC | 478 – 1034 (557) | NC002924 |

From Ashimoto et al.^16^, Manufacter: Oligos (Wilsonville, USA). Amplifications were performed in a PTC-100 thermocycler (MJ Research Inc., Boston, MA), following this program: initial denaturation under 95^o^C for 2 minutes, followed by 36 cycles of denaturation under 95^o^C for thirty seconds, annealing under 60^o^C for 1 minute, extension under 72^o^C for 1 minute and final extension under 72^o^C for 2 minutes.
