# Supplementary material for: Coronary atherosclerosis and periodontitis have similarities in their clinical presentation
Source: Front Oral Health. 2024 Jan 16;4:1324528. doi: 10.3389/froh.2023.1324528 (PMC10825671; doi:10.3389/froh.2023.1324528)
Supplement: Supplementary file 2 [file Table2.docx]

**Supplementary Table 2. Demographic and Clinical data from subjects with collected atheroma**

| Variable | Value |
| --- | --- |
| Male | 17 (56.7%) |
| Age (years) | 71.17 (±7.45) |
| Samples | 14 Left Carotid  6 Right Carotid  10 Coronaries |
| Number of Teeth | > 6 teeth (100); 2 subjects with no molars |
| Bleeding on Subgingival Biofilm Collection (per subject) | 30 (100%) |
